# Wilberforce-like Larmor Magnetic Moment and Spin Precession

**DOI:** 10.3390/e26090736

**Published:** 2024-08-29

**Authors:** Ferenc Márkus, Katalin Gambár

**Affiliations:** 1 Department of Physics, Budapest University of Technology and Economics, Műegyetem rkp. 3, H-1111 Budapest, Hungary; markus.ferenc@ttk.bme.hu; 2 Department of Natural Sciences, Institute of Electrophysics, Kálmán Kandó Faculty of Electrical Engineering, Óbuda University, Tavaszmezőu. 17, H-1084 Budapest, Hungary; 3 Department of Natural Sciences, National University of Public Service, Ludovika tér 2, H-1083 Budapest, Hungary

**Keywords:** Wilberforce pendulum, cyclotron frequency, Larmor precession, Langevin diamagnetism, magnetic moment and spin waves, coupled interactions, Lagrangian, loss of coherence

## Abstract

In a Wilberforce pendulum, two mechanical oscillators are coupled: one pertains to the longitudinal (tension) motion and the other to the rotational (twisting) motion. It is shown that the longitudinal magnetic moment of circular currents, and similarly the magnetic moment of a spin-chain, can exhibit a Wilberforce-like vibration. The longitudinal oscillation is related to the Langevin diamagnetism, while the twisting motion is superimposed on the magnetic moment and spin precession. The calculations show that the coupling term is nonlinear in this (longitudinal) vibrating and (magnetic moment) precession system. By increasing the strength of the coupling we arrive at a spectrum, where further vibrational modes can be associated with the rotation of the precession. This means that the extent of the change in coherence can be demonstrated. Since the coupling strength can be different due to local effects, this can be an important factor from the point of view of signal propagation and in preserving signal shapes. The amount specifying the dissipation is introduced to express the degree of deviation. A relationship exists between the parameter characteristic of the coupling strength and how its quantity influences decoherence and dissipation.

## 1. Introduction

We consider an electron chain along the *x* direction, where the electrons move around in closed loops with a cyclotron frequency of $\omega_{c}$ and their magnetic moment precess with a Larmor frequency of $\omega_{p}$ [1,2]. The applied external magnetic field of *B* is parallel to the axis *x*, and the orientation of magnetic moments of the rotating electrons is also parallel to this direction.

**Figure 1:**
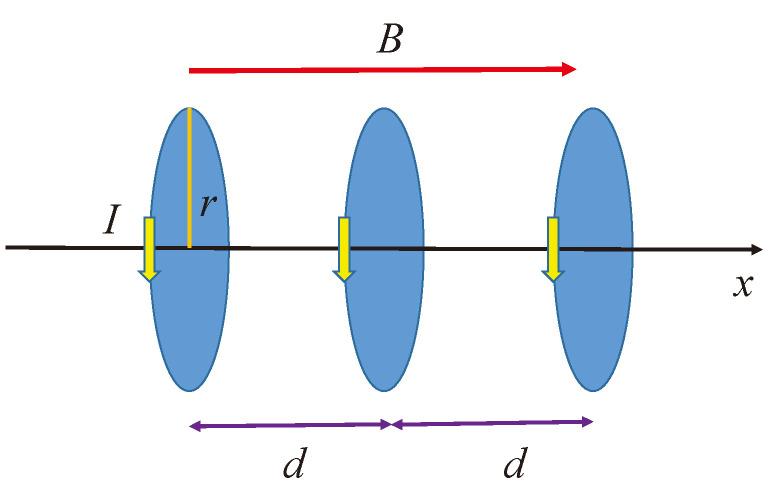
The initial arrangement of the current loops.

Due to the magnetic field created by the rotating electrons, these current loops of *I* interact with each other, which causes a distance of *d* between the loops to vary continuously within the chain. The physical arrangement of the loops and the magnetic field are shown in Figure 1.

In the present model, we assume coupling between the translational vibration and the Larmor precessional movement. Our goal is to highlight the specifics of the movement and how it might be relevant to physical systems. In the linear Wilberforce pendulum model the translational and rotational vibrations are coupled. Our considerations are based on this model [3,4,5]. The idea is to formulate the movement of current loops by drawing an analogy to this oscillation. Mathematically, the difference is that the coupling between translational and rotational motion will be nonlinear. As a result, depending on the strength of the coupling, additional excitations appear in addition to the main vibration modes. If the coupling strength changes at a specific point in the current loop chain, the coherence of the vibration is lost. This is an important effect in signal propagation. It means that the transmission of the vibration becomes decoherent. Moreover, if the distortion is not just a phase shift, then dissipation can occur. An important result of this work is that we can quantify this dissipation. Therefore, it is bringing us closer to an understanding of the apparent irreversibility [6].

The sections of the article are as follows. In Section 2 we discuss the necessary background phenomena: the Wilberforce pendulum, Langevin diamagnetism, and Larmor precession. The perturbation of the magnetic field, and its interaction with the translation, are examined in Section 3. The precession of the circular current magnetic moment and the interaction energy expression are calculated in Section 4. The Lagrangian function of the constructed model is described in Section 5. In Section 6, we derive the equations of motion and solve them numerically for different coupling coefficients. The dependence of the coupling strength of the longitudinal vibration displacement and the vibrating change of the precession angle can be observed. In Section 7, we discuss how the magnetic moment associated with the spin behaves similarly to the magnetic moment of the current loop, thus, the results can be extended to electron spin as well. The issue of coherence loss and dissipation is discussed in Section 8. Studying the spectra, we will show that the degree of decoherence and the excitations that appear by increasing the coupling strength can be linked. In Section 9 we give a mathematical procedure for calculating an exact, numerical formulation of the degree of decoherence and dissipation from the spectra. Quantifying these quantities can be defined in this way and can also answer the extent to which the transmitted signal, distorted due to the change in coupling, can be reconstructed. In the last Section 10, we give a summary of the results of the article.

## 2. Backgrounds of the Physical Model

We intend to build a model that includes the mutual influence of the current loops moving through the chain. The suitable physical movements for this are the Wilberforce pendulum, Langevin diamagnetism, and Larmor precession. In this section, we are reviewing these movements.

### 2.1. The Wilberforce Pendulum

The Wilberforce pendulum [3,4,5] consists of a point-like body with a mass at the end of a massless spring that can vibrate longitudinally in the *z*-direction and torsionally. Longitudinal motion is described in the usual way in the theory. The twisting–rotating motion of the spring occurs around the *z*-axis. The envelope of the spring is a cylinder, i.e., the spring cannot break out laterally. The Lagrangian $\tilde{L}$ reflects the conservation of mechanical energy. The coupling term transfers energy between the longitudinal translational and torsional motion. The mathematical relationship between the angle of torsion and displacement is

(1) φ(z)∼z,

so, the interaction term is proportional to the product of the torsion angle and the displacement:

(2) φz.

Applying the kinetic and potential energy terms both for the translational and the precession motion, and for the coupling, we obtain the Lagrangian:

(3) $$\tilde{L}=\frac{1}{2}m{\dot{z}}^{2}-\frac{1}{2}kz^{2}+\frac{1}{2}\Theta {\dot{\varphi }}^{2}-\frac{1}{2}D\varphi^{2}+\frac{1}{2}\epsilon \varphi z.$$

Here, the mass of the body is *m*, the spring constant is *k*, the moment of inertia is Θ, the torsion constant is *D*, and the coupling parameter is ϵ. The equations of motion are the Euler–Lagrange equations

(4) $$0=m\ddot{z}+kz-\frac{1}{2}\epsilon \varphi$$

and

(5) $$0=\Theta \ddot{\varphi }+D\varphi +\frac{1}{2}\epsilon z.$$

Here, it is useful to introduce the translational and rotational angular frequencies

(6) $$\omega_{z}^{2}=\frac{k}{m};\;\;\;\;\;\;\omega_{\varphi }^{2}=\frac{D}{\Theta },$$

by which we can express the frequency of the normal modes:

(7) $$\omega_{1}^{2}=\frac{1}{2}\left(\omega_{z}^{2}+\omega_{\varphi }^{2}+{\left[{\left(\omega_{z}^{2}-\omega_{\varphi }^{2}\right)}^{2}+\frac{\epsilon^{2}}{m\Theta }\right]}^{1/2}\right)$$

and

(8) $$\omega_{2}^{2}=\frac{1}{2}\left(\omega_{z}^{2}+\omega_{\varphi }^{2}-{\left[{\left(\omega_{z}^{2}-\omega_{\varphi }^{2}\right)}^{2}+\frac{\epsilon^{2}}{m\Theta }\right]}^{1/2}\right).$$

The exact solution can be achieved using the normal mode technique [4]. Applying the initial conditions

(9) $$z\left(0\right)=0;\;\;\;\dot{z}\left(0\right)=0;\;\;\;\varphi \left(0\right)=\varphi_{0};\;\;\;\dot{\varphi }\left(0\right)=0,$$

the solutions are

(10) $$\begin{array}{ccc} z(t) & = & \frac{z_{0}}{\omega_{1}^{2}-\omega_{2}^{2}}\left[\left(\omega_{1}^{2}-\omega_{\varphi }^{2}\right)\cos\;\omega_{1}t-\left(\omega_{2}^{2}-\omega_{\varphi }^{2}\right)\cos\;\omega_{2}t\right]\\ & - & \frac{2\Theta \varphi_{0}}{\epsilon }\frac{1}{\omega_{1}^{2}-\omega_{2}^{2}}\left(\omega_{1}^{2}-\omega_{\varphi }^{2}\right)\left(\omega_{2}^{2}-\omega_{\varphi }^{2}\right)\left(\cos\;\omega_{1}t-\cos\;\omega_{2}t\right), \end{array}$$

and

(11) $$\begin{array}{ccc} \varphi (t) & = & \frac{\epsilon z_{0}}{2\Theta }\frac{1}{\omega_{1}^{2}-\omega_{2}^{2}}\left(\cos\;\omega_{1}t-\cos\;\omega_{2}t\right)\\ & + & \varphi_{0}\frac{1}{\omega_{1}^{2}-\omega_{2}^{2}}\left[\left(\omega_{1}^{2}-\omega_{\varphi }^{2}\right)\cos\;\omega_{1}t-\left(\omega_{2}^{2}-\omega_{\varphi }^{2}\right)\cos\;\omega_{2}t\right]. \end{array}$$

These exact solutions can be obtained because of the linear coupling. Naturally, the coupling strength cannot only be linear. In the example discussed later in the article, we numerically solve the motion of the Wilberforce pendulum and perform analyses on the nonlinear regime.

### 2.2. Langevin Diamagnetism

Classically, an external magnetic field acts on the electrons in materials, forcing them into circular current loops. These microscopic currents from the orbitals of bounded electrons and their related magnetic field oppose the changing external generator field of $B^{\prime }$. This effect is known as Langevin diamagnetism [1]. We need to calculate the magnetic moment of these induced microscopic currents for further aims. We start with the Faraday’s induction law

(12) $$\oint Eds=-\int \frac{\partial B^{\prime }}{\partial t}dA$$

Assuming a circular motion, the magnitude of the electric field *E* is

(13) $$E=-\frac{r}{2}\frac{dB^{\prime }}{dt},$$

where *r* is the radius of the loop. We consider a spatially uniform magnetic field. The electric field of *E* exerts a force on the *q* electron charge, resulting in a torque of r×qE. We apply the angular momentum theorem

(14) $$\frac{dJ}{dt}=qrE.$$

(15) $$E=\frac{1}{qr}\frac{dJ}{dt}$$

where *J* is the orbital angular momentum.

(16) $$\frac{dJ}{dt}=-\frac{qr^{2}}{2}\frac{dB^{\prime }}{dt}$$

Integrating over the required time interval reveals a change in the angular momentum due to $B^{\prime }$

(17) $$\Delta J=-\frac{qr^{2}}{2}B^{\prime }.$$

Applying the relation between the magnetic momentum μ and the angular moment

(18) $$\mu =\frac{q}{2m}J,$$

we can formulate the connection between the induced moment $\mu^{\prime }$ and $B^{\prime }$

(19) $$\mu^{\prime }=\frac{q}{2m}\Delta J=-\frac{q^{2}r^{2}}{4m}B^{\prime }.$$

This obtained relation is the Langevin diamagnetism formula. The main point is that the changing magnetic field regulates the induced magnetic moment.

Note that some electrically conductive materials, such as tungsten, aluminum, and magnesium, are paramagnetic. In these materials, the paramagnetic effect can exceed the Langevin diamagnetic effect. Therefore, the most significant influence is expected in diamagnetic conductors such as copper, silver, graphite, and bismuth. In the present work, we focus on net diamagnetic materials.

### 2.3. Magnetic Moment Precession

The torque on the current loop due to a magnetic moment μ in magnetic field of B is

(20) τ=μ×B

The circular motion of the charged particles has angular momentum. The magnetic moment and the angular momentum are proportional

(21) μ=γJ,

where γ is the gyromagnetic ratio. The equation of motion given by the angular momentum theorem is

(22) $$\;\frac{dJ}{dt}=\gamma J\times B.$$

On the other hand

(23) $$\frac{dJ}{dt}=\omega_{p}\times J,$$

where $\omega_{p}$ is the Larmor frequency. Comparing Equations (22) and (23) we can obtain

(24) $$\omega_{p}=-\gamma B,$$

That is, the change in the magnetic field causes the change in the angular velocity of the precession.

In the following, we will show that the above theory can be applied to a system with a magnetic moment placed in a magnetic field.

## 3. Perturbational Magnetic Field and the Related Longitudinal Motion

**Figure 2:**
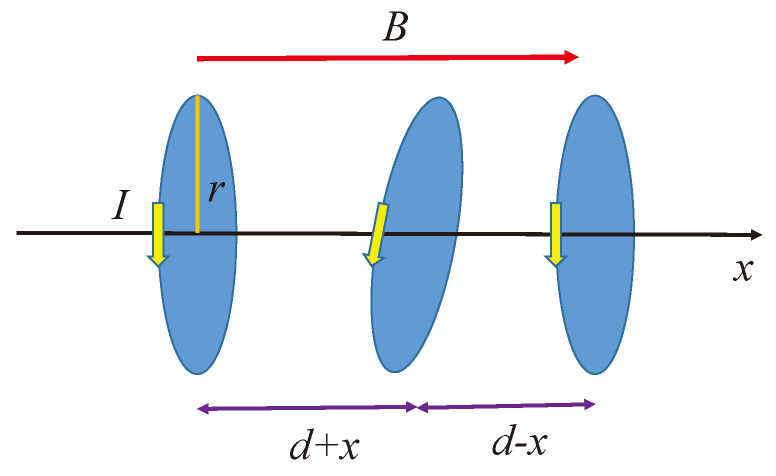
The small dispalcement of the current loops.

We consider three current loops, each carrying a current *I* originating from ions whose orbiting electrons will generate Langevin diamagnetism − in a homogeneous longitudinal direction magnetic field *B*. The currents relate to a cyclotron frequency $\omega_{c}=Bq/m$ by $I=q\omega_{c}/2\pi$, as Figure 1 shows. This initial arrangement appears to be cylindrically symmetric, but the correct assumption is that the current loops are disordered and we must try to align them in the direction of the magnetic field when the magnetic field is turned on. Thus, the precession movement starts promptly. Due to the additional magnetic field created by the rotating electrons, these current loops interact with each other, which causes the distance between the loops to change continuously within the chain. We assume that the middle loop can freely move longitudinally in the direction of the rotational axis with a small *x* displacement, as Figure 2 shows. The other two loops are fixed.

First, we need to obtain the change in energy due to this perturbation, so we calculate the appearance of an additional magnetic field at the coordinate of the middle loop. First, we express the total magnetic field from the external field and the three loops as

(25) $$B\left(x\right)=B+\mu_{0}\frac{q\omega_{c}R^{2}}{4\pi }\left(\frac{1}{{\left(R^{2}+{\left(d+x\right)}^{2}\right)}^{3/2}}+\frac{1}{R^{3}}+\frac{1}{{\left(R^{2}+{\left(d-x\right)}^{2}\right)}^{3/2}}\right).$$

Now, we take the Taylor series expansion for small x≪d values, by which we can approximate the B(x) for the middle loop

(26) $$B\left(x\right)=B+\mu_{0}\frac{q\omega_{c}R^{2}}{4\pi }\left(\frac{1}{R^{3}}+\frac{2}{{\left(R^{2}+d^{2}\right)}^{3/2}}\right)+\mu_{0}\frac{q\omega_{c}R^{2}}{4\pi }\frac{3(4d^{2}-R^{2})}{{\left(R^{2}+d^{2}\right)}^{7/2}}x^{2},$$

where the second term on the right-hand side, $B_{0}$, is the constant magnetic field from the loops, and the third term, $B^{\prime }\left(x\right)∼\;x^{2}$, is the perturbation due to the motion at the location of the middle loop. The perturbed magnetic term is proportional to $x^{2}$, thus

(27) $$\mu^{\prime }\left(x\right)=-\mu_{0}\frac{q^{3}\omega_{c}R^{4}}{16\pi m}\frac{3(4d^{2}-R^{2})}{{\left(R^{2}+d^{2}\right)}^{7/2}}x^{2}.$$

The energy contribution of the interaction with the middle loop is

(28) $$\Delta E_{p}\left(x\right)=-\mu^{\prime }\left(x\right)\;B\left(x\right)\;\;\;=\;\;\;\frac{1}{2}Kx^{2}+\frac{1}{4}Lx^{4}.$$

Here, $\mu^{\prime }$ depends on the spatial coordinate, *x*. The parameters *K* and *L* can be calculated from the products of Equations (26) and (27). The parameters are positive if d>R/2. Considering the typical atomic radius and the usual spacing between atoms in solids, this is satisfied. If we restrict our examination to the first approximation we can write the energy of the loop as a quadratic potential energy

(29) $$\Delta E_{p}\left(x\right)\;\;\;∼\;\;\;\frac{1}{2}Kx^{2}.$$

The loop, which is the charged particle itself, has a mass *m*. If $\dot{x}$ means the perturbational velocity of the single loop, then its kinetic energy is

(30) $$E_{k}\left(x\right)=\frac{1}{2}m{\dot{x}}^{2}.$$

## 4. Perturbed Magnetic Moment Precession Motion

The additional magnetic moment $\mu^{\prime }$ interacts with the magnetic field B. Then, an additional torque $\tau^{\prime }=\mu^{\prime }\times B$ contributes to the torque, τ, in Equation (20). This results in a precession change by an angle of φ which appears as a continuous perturbation. We add this modifying effect to the description.

We consider the precession angular velocity $\omega_{p}$, superposing an additional $\dot{\varphi }$ generated from the longitudinal motion-related magnetic interaction, i.e., the total angular velocity of the charged particles is $\omega \left(t\right)=\omega_{p}+\dot{\varphi }$. The angular velocity of the Larmor frequency continuously changes. This is because the relevant magnetic moment orientation of the interacting loops causes a magnetic moment—a magnetic moment interaction which generates an angle-dependent intrinsic torque phase. The measure of this torque is proportional to the sine of the angles of the magnetic moments. An initial approximation for the small angles (sinφ∼φ) of the torque can be expressed using

(31) τ=−Dφ,

where *D* is the torsion constant. This provides a simple analogy for the standard Wilberforce pendulum.

The rotational energy of the loop is

(32) $$E_{k}\left(t\right)=\frac{1}{2}\Theta \omega^{2}\left(t\right)=\frac{1}{2}\Theta {\left(\omega_{p}+\dot{\varphi }\right)}^{2},$$

where Θ is the moment of inertia of the precessing loop.

The related potential energy is

(33) $$E_{p}\left(\varphi \right)=\frac{1}{2}D\varphi^{2}.$$

Following the relations in Equations (24) and (26), we can recognize a twisting–vibrating motion superimposed on the Larmor precession

(34) $$\omega \left(t\right)=\omega_{p}+\dot{\varphi }\left(t\right)=-\gamma B\left(x\right).$$

The Larmor frequency $\omega_{p}$ pertains to the static magnetic field ($B_{0}+$ being the external field), and $\dot{\varphi }$ is a consequence of $B^{\prime }\left(x\right)$. Applying Equation (26), we can deduce a relation between $\dot{\varphi }$ and the displacement *x*:

(35) $$\dot{\varphi }\left(t\right)=-\gamma B^{\prime }\left(x\right)∼-x^{2}\left(t\right).$$

This mathematical relationship represents a nonlinear coupling between the two physical quantities. We need to formulate the energy term of the coupling interaction. Similar to the interaction in Equations (1) and (2), it seems a relevant choice to follow this method. The coupling interaction is a product of the two deduced variables $\dot{\varphi }$ and $x^{2}$:

(36) $$E_{\mathrm{int}}=R\dot{\varphi }\left(t\right)x^{2}\left(t\right).$$

The previous relation holds to a constant coupling factor, *R*. The entire energy of the system is the sum of the terms in Equations (30), (32), and (36). It is worth emphasizing that the total energy of the system is conserved. The coupling term generates the energy transfer between the longitudinal oscillation and the torsional rotation. Nevertheless, mechanical energy is not lost. However, with the change in coupling, such events occur where the vibrations become decoherent and dissipative relative to the initial state. In the following, we develop our analysis in this direction.

## 5. Lagrangian and Hamiltonian

By applying the results of the previous chapter, we are in a relatively simple situation in terms of expressing the Lagrange function. In fact, we need to add the corresponding energy terms as shown in Equation (37). The Lagrangian, $\bar{L}$, for the presently discussed case, has a usual form, however, the interacting term differs from the bilinear form in the Wilberforce pendulum, since it follows from Equations (35) and (36). Since the Lagrangian does not depend explicitly on time (and does not contain complex/non-Hermitian terms), we can be sure that the total energy is conserved

(37) $$\bar{L}=\frac{1}{2}m{\dot{x}}^{2}-\frac{1}{2}Kx^{2}+\frac{1}{2}\Theta {\left(\omega_{p}+\dot{\varphi }\right)}^{2}-\frac{1}{2}D\varphi^{2}+R\dot{\varphi }x^{2}.$$

The first two terms pertain to the compression caused by the Langevin diamagnetism and the third and fourth terms describe the precession motion. The last term is the interaction between them. This can be deduced from Equation (35). The product expresses the mutual dependence of the two phenomena. It also means a contribution to the energy. The parameter *R* describes the coupling strength between the translational displacement and rotating vibration.

To construct the Hamiltonian, first, we must calculate the canonical quantities. The conjugated variable of *x* is

(38) $$p_{x}=\frac{\partial \bar{L}}{\partial \dot{x}}=m\dot{x},$$

and for φ is

(39) $$p_{\varphi }=\frac{\partial \bar{L}}{\partial \dot{\varphi }}=\Theta \dot{\varphi }+Rx^{2}.$$

Then the Hamiltonian is

(40) $$H=p_{x}\dot{x}+p_{\varphi }\dot{\varphi }-\bar{L}=\frac{1}{2}m{\dot{x}}^{2}+\frac{1}{2}Kx^{2}+\frac{1}{2}\Theta {\left(\omega_{p}+\dot{\varphi }\right)}^{2}+\frac{1}{2}D\varphi^{2}.$$

Notice that the total energy of this Wilberforce system is independent of the coupling. This does not mean that the coupling does not carry energy, but that the entirety of the energy can be determined from the initial translational and precessional energy. This gives the same energy value regardless of the coupling strength for the same initial conditions.

## 6. Equations of Motion

Applying variational calculus for variables *x* and φ we obtain the equations of motion in the form of Euler–Lagrange equations. These are two, coupled, ordinary nonlinear differential equations

(41) $$0=m\ddot{x}+Kx-2Rx\dot{\varphi }$$

and

(42) $$0=\Theta \ddot{\varphi }+D\varphi +2Rx\dot{x}.$$

The nonlinearity arises from the coupling terms, which are the third terms in both of these equations. The structure of these coupled differential equations is similar to the Wilberforce pendulum in Section 2.1. The key difference here is in the nonlinear coupling. The solution can be obtained through numerical calculations. To find the solutions we need to handle the parameter set. It is an obvious choice to take a unit mass of m=1 kg and a unit moment for inertia of Θ=1 kg m^2^. Thus, we are left with three freely adjustable parameters. We compare solutions for three different sets of parameters. We fix the parameters K=10 N/m and D=90 Nm and we change the coupling parameter *R*. (The chosen values of the parameters do not affect this article’s conclusions. The parameters can be scaled as desired.) The initial conditions are x(0)=1 m; $\dot{x}\left(0\right)=0$ m/s; φ(0)=0 rad; and $\dot{\varphi }\left(0\right)=-2$ rad/s. We use these initial conditions throughout the Wilberforce pendulum calculations.

(a) In the first case, we chose R=0.2 Ns/m. This is the weak coupling case.

**Figure 3:**
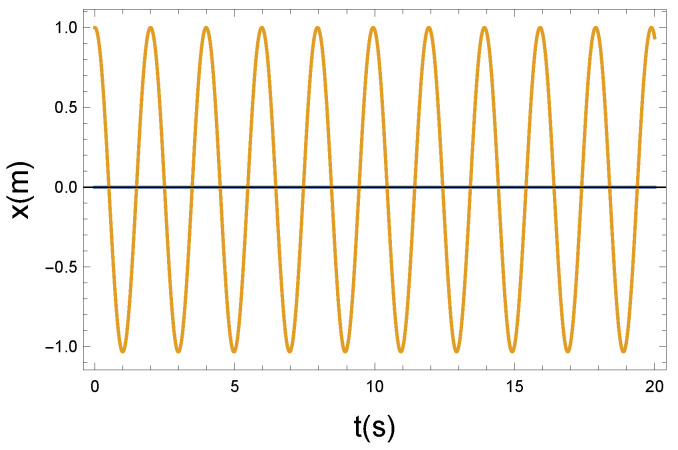
The longitudinal vibrating displacement *x* as a function of time *t*. Due to the nonlinear x−φ coupling, the plotted x(t) solution function is almost periodic. Parameters: K=10 N/m, D=90 Nm, and R=0.2 Ns/m. The small parameter of *R* pertains to a weak coupling.

The local vibrating displacement of x(t) in Figure 3 is superposed on the longitudinal translational motion of the magnetic moment. The motion looks like a longitudinal compression wave with small amplitude displacement. The speed of the wave is $v_{0}$. This constant velocity has no essential physical role in the present examination.

**Figure 4:**
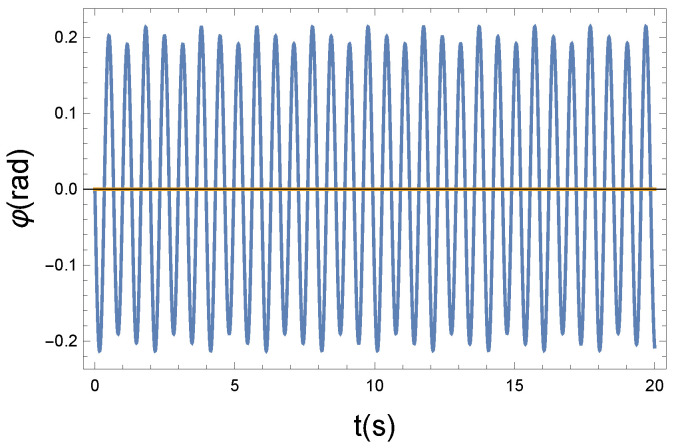
The Larmor precession is influenced by the longitudinal vibrating displacement via the Langevin diamagnetism effect. The vibrating change of the precession angle is described using the function φ(t). Due to the nonlinear x−φ coupling, the plotted φ(t) solution function is almost periodic. Parameters: K=10 N/m, D=90 Nm, and R=0.2 Ns/m. The small parameter of *R* pertains to a weak coupling.

The φ(t) function that modifies the precession has an almost sinusoidal shape, so as a consequence of the weak coupling, the precession of the magnetic moments remains coherent. It seems apparent from Figure 3 and Figure 4 that the coupling is weak. Practically, the longitudinal and precessional vibrations are independent of each other. In the case of R=0, the decoupling is perfect.

**Figure 5:**
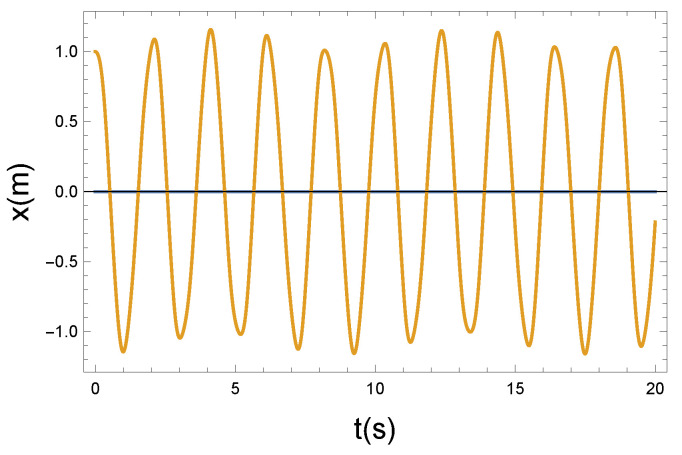
The longitudinal vibrating displacement *x* as a funtion of time *t*. Due to the nonlinear x−φ coupling, the plotted x(t) solution function is not periodic. Parameters: K=10 N/m, D=90 Nm, and R=1.4 Ns/m.

(b) We can turn on a higher coupling by increasing the parameter of *R*. Now, let us consider a seven-times stronger coupling with a strength of R=1.4 Ns/m. Figure 5 shows the evolution of the time dependence of the longitudinal vibrating displacement x(t) over time. Comparing it with the plot of the weakly interacting case in Figure 3, we do not see radical change, however, we can recognize amplitude waving and a small time shift. It looks similar to a modulation envelope. Since the longitudinal vibration motion is not the most essential point in the magnetic moment transfer, these modifications may be negligible.

**Figure 6:**
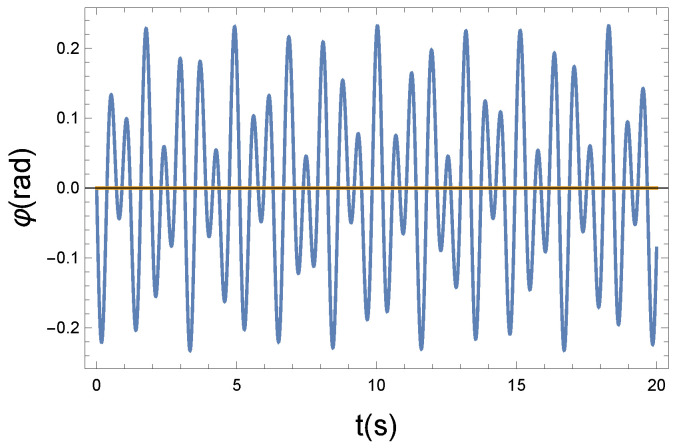
The Larmor precession is influenced by the longitudinal vibrating displacement via the Langevin diamagnetism effect. The vibrating change of the precession angle is described by the function φ(t). Due to the nonlinear x−φ coupling, the plotted φ(t) solution function is not periodic. Parameters: K=10 N/m, D=90 Nm, and R=1.4 Ns/m.

However, the temporal change in the angle of φ is much larger. The undulating change in the amplitude is evident from Figure 6. Comparing this graph with Figure 4, the loss of coherence is obvious.

Figure 6 shows the time dependence of the phase vibration, φ(t). This phase vibration is superposed on the Larmor precession modifying its angular velocity $\omega_{p}$. The graph reflects well that the continuous amplitude and phase change destroy the coherence. We can conclude that due to the nonlinear and asymmetric coupling, the obtained graphs are slightly different from the usual Wilberforce pendulum shape, however, the main characteristics are essentially the same.

**Figure 7:**
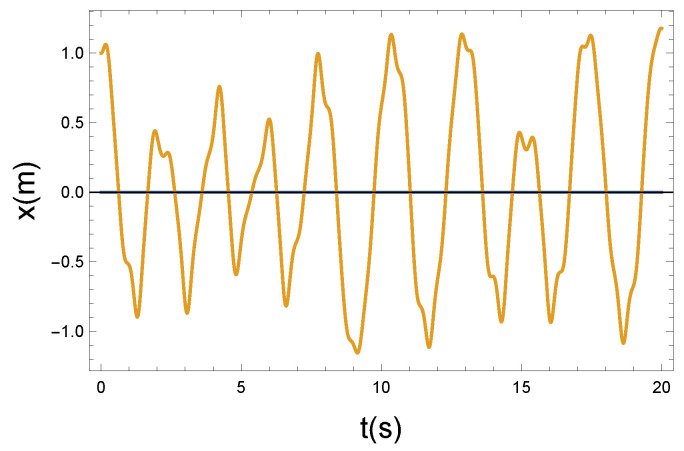
The longitudinal vibrating displacement *x* as a funtion of time *t*. Due to the nonlinear x−φ coupling, the plotted x(t) solution function is rather incoherent. Parameters: K=10 N/m, D=90 Nm, and R=5 Ns/m.

**Figure 8:**
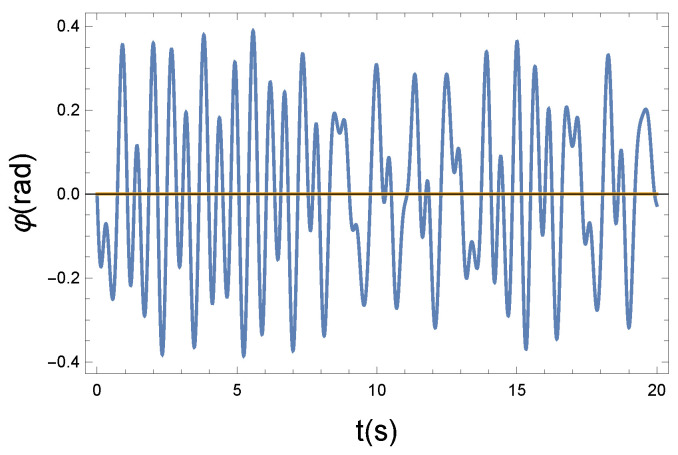
The Larmor precession is influenced by the longitudinal vibrating displacement via the Langevin diamagnetism effect involving random-like effects. The vibrating change of the precession angle is described by the function φ(t). Due to the nonlinear x−φ coupling the plotted φ(t) solution function is rather incoherent. Parameters: K=10 N/m, D=90 Nm, and R=5 Ns/m.

(c) The third case pertains to the strong coupling with R=5 Ns/m. As shown in Figure 7 and Figure 8, both the displacement and the precession angles have a peculiar drag on the oscillating motion. Excessive nonlinear coupling even eliminates periodic behavior. One might think that if this behavior occurs, its technical applicability (e.g., signal transfer and reconstruction) is doubtful. In this case, we certainly cannot talk about coherent behavior. It mostly resembles a scattered, chaotic phenomenon.

## 7. Contribution of Spin Magnetic Effect

From a simple and practical point of view, we represented the magnetic moments using the circular currents, as discussed previously. Thus, we did not deal with what creates the current loop, because just the existence of the magnetic moment is important in the present results. In the case of current loops, the magnetic field and its change can be easily demonstrated, as shown in Equations (25) and (26). Of course, these current loops and magnetic moments can correspond the electron currents in the most obvious way. Now, let us ignore the current loop and only consider the presence of a magnetic moment. Electrons have spins that also generate a magnetic moment:

(43) $$\mu_{S}=-\frac{e}{2m}gS,$$

where *e* is the charge and *m* is the mass of the electron, *g* is the giromagnetic factor (*g* = 2.0023 for the free electron), and *S* is the quantized intrinsic angular momentum spin. The quantized angular momenta has a similar structure

(44) $$S=\sqrt{s(s+1)}\hbar ,$$

where *s* is the quantum number and *ℏ* is the reduced Planck constant. This quantum number for an electron is s=1/2.

Due to quantum mechanical reasons, the total electron spin can only have a value of 1/2ℏ in the direction of the magnetic field. However, this fact does not change the description of the precession of the current loop. The projection in the direction of the magnetic field is constant; if the magnetic field changes with time, then the angular velocity of the precession changes with time, and similar to the magnetic moment of the loop currents, our findings on current loops can be fully transferred.

## 8. Dissipation, Loss of Coherence

There is no energy loss in a Wilberforce pendulum, which is the coupling of a magnetic moment and a longitudinal motion. Conservative forces are at work, and there is a coupling between the two movements. Since the entire energy of the system is constant, it is not irreversible in a thermodynamic sense. At the same time, according to nonlinear coupling, it is irregularly transferred back and forth between the two types of movement. Despite the entire energy of the system being conserved, this chaotic behavior causes the movements to be incoherent. When the magnetic moment transfers, the spin wave becomes incoherent. The coherence loss that appears in this way can be important in signal transmission via spins or via spin waves [7,8,9,10,11,12].

**Figure 9:**
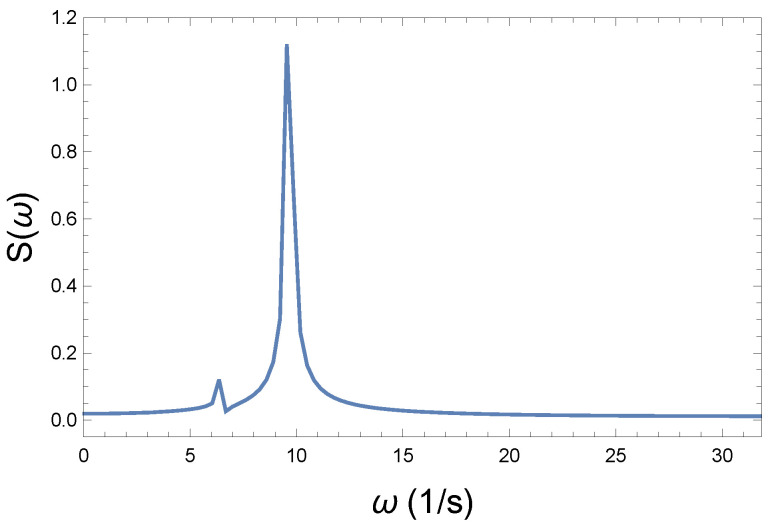
Spectrum of the weak coupling R=0.2 Ns/m. The sharp peak represents the frequency of the coupling-free R=0 Ns/m. Normal mode. The broadening of the peak is a natural consequence of the nonlinear coupling. The smaller peak comes from the coupling effect.

**Figure 10:**
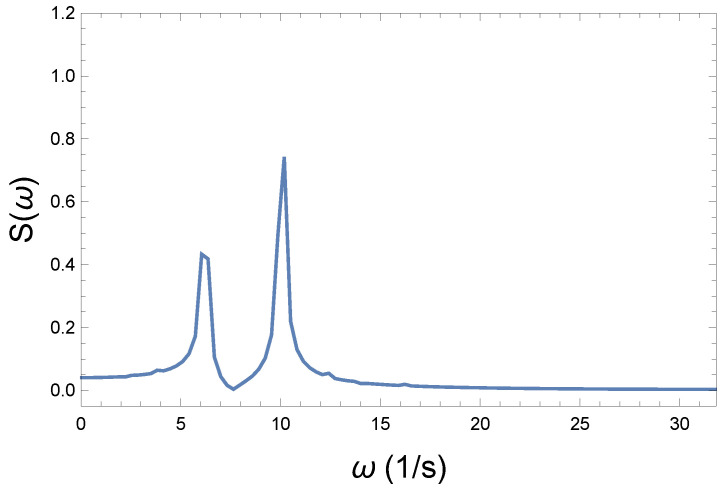
Spectrum of the medium coupling R=1.4 Ns/m. The ratio of the normal modes changes. The higher peak decreases spectacularly, while the smaller one is of the same magnitude as the other one. At the same time, the appearance of lower frequencies is also visible.

**Figure 11:**
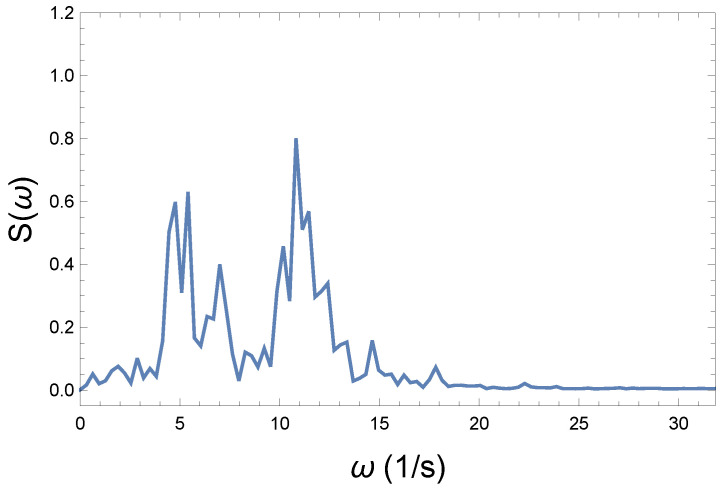
Spectrum of the strong coupling R=5 Ns/m. Excitations appear throughout the entire frequency range. The normal modes of the initial weak coupling can hardly be identified.

Now, the question is, how can we express the loss of coherence, the increase in chaoticity, and ultimately the measure of irreversibility? We believe that in the Wilberforce system, precession plays the more important role in information transmission. Figure 4, Figure 6, and Figure 8 show phase changes over time at different coupling strengths. Although the difference is obvious, a mathematical tool is still needed to give the correct measure. From the comparison, we can see that as the coupling strength increases, more and more new frequencies, different from the "base" frequency, appear. For this reason, it is advisable to recognize those frequencies that appear during the precession in each case. This shows the displayed frequency values using the natural proportions of the spectral distribution. The idea is therefore to produce the spectra belonging to the precession. From their comparison, we can draw further conclusions. By comparing the spectra, we can immediately establish important physical conclusions about the process. That is why we plot in Figure 9, Figure 10 and Figure 11 the spectra’s (spectral densities; the absolute values of Fourier transforms in Figure 4, Figure 6, and Figure 8) for all three couplings.

The spectrum shown in Figure 9 belongs to the weak coupling. Without coupling, the circular frequency for precession from Equation (5) is

(45) $$\omega_{0}=\sqrt{\frac{D}{\Theta }}=9.5\;\mathrm{rad}$$

value. This pertains to the dominant peak in Figure 9. However, it can be seen that there is another angular frequency. This tiny peak close to it is due to the coupling. Now, let us assume that this contribution exists, but it is not significant.

The spectrum of the medium-strength coupling is shown in Figure 10. Comparing the larger vs. the tiny peaks, we see that the main normal frequency decreases, while the frequency belonging to the tiny peak already appearing in Figure 9 increases significantly, i.e., their ratio decreases. Furthermore, the appearance of lower frequencies is also noticeable. At the same time, the change still seems quite smooth.

The spectrum of the strong coupling in Figure 11 clearly shows the significant changes due to coupling strength. Smaller excitations appear throughout the entire frequency range. The original spectrum becomes fragmented due to further excitations. The characteristics of the initial weak coupling spectrum disappear almost completely.

**Figure 12:**
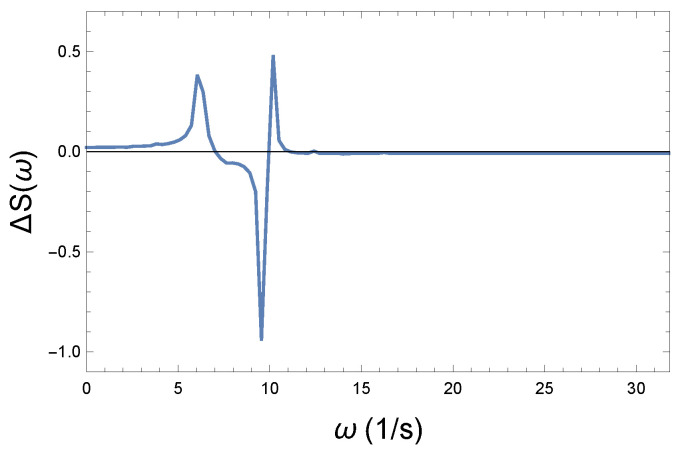
The difference between the spectrum of medium and weak couplings. Frequencies with positive values are those that appeared during the strengthening of the coupling. These contribute to the loss of coherence and irreversibility.

We can see how new excitations appear in the spectrum compared to the initial (weak) coupling. Let us start from the assumption that the physical system is characterized by a state of weak coupling, however, at a given location the coupling strength has changed to a different value. In our example, consider a coupling of medium strength. By subtracting the initial spectrum from the latter, the appearing frequencies in the positive upper range become visible, as shown in Figure 12.

## 9. Measure of Decoherence

In what follows, we start from the fact that within the examined system, there can be places where the coupling strength is different at any point in the space. The physical reason will not be detailed now, we will only assume that it increases slightly compared to the value characteristic of the process. As discussed in the previous chapter, as the coupling strength increases, as a consequence of the nonlinear coupling, additional frequencies appear compared to the starting frequencies. The stronger the coupling, the more modes appear. Since this differs from the initial frequency spectrum, it also means a change in the coherence of the vibration. If this occurs in a physical signal transmission, it can cause substantial distortion. Therefore, the change appearing in the spectrum should be mapped in detail.

**Figure 13:**
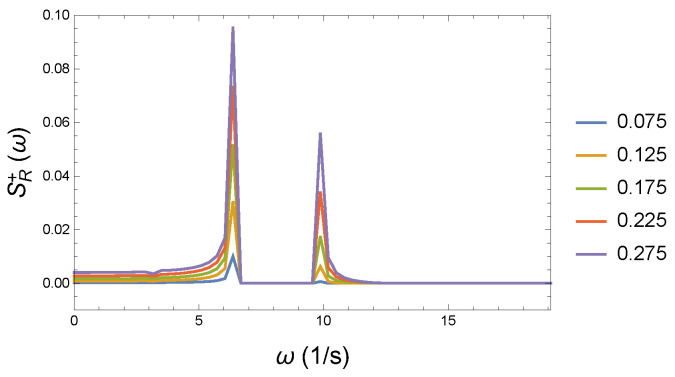
The plot of the positive values of the difference in the spectra at different strengths of couplings *R* and the reference spectrum $R_{0}$. The apparent additional excitations contribute to the loss in coherence and irreversibility. The reference coupling is $R_{0}=0.05$ Ns/m. The color legends pertain to the relevant coupling parameter *R*.

We assume a coupled system that can be specified with the following physical parameters: K=10 N/m, D=90 Nm, and $R_{0}=0.05$ Ns/m. We treat this system as the basic starting system. After that, we increase the coupling parameter, demonstrating the deviation and the distortion inside the system. These parameters are R= 0.075; 0.125; 0.175; 0.225; and 0.275 Ns/m, respectively. We choose small deviations so that we can track subtle changes. We calculate the corresponding spectra for all parameters as plotted in Figure 9, Figure 10 and Figure 11. The appearing new modes related to the increased coupling can be easily visualized by subtracting the two spectra from each other, as depicted in Figure 12. Since we are only interested in new excitations, it is sufficient to consider the set of positive values of the spectrum. The resulting $S_{R}^{+}\left(\omega \right)$ spectrum can be seen in Figure 13. The circular frequency values are plotted on the horizontal axis as a function of ω. The color legends belong to the different couplings. The figure shows that by increasing the coupling strength, both lower and higher frequencies compared to the “main excitations” (ω∼6.5–9.5 1/s) appear. It is quite natural that the frequency image is not symmetrical. Rather, lower frequencies dominate. The rate of growth is monotonic and smooth.

**Figure 14:**
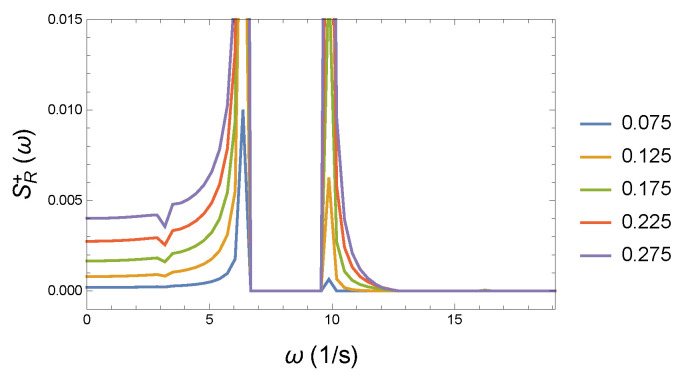
Enlarged section of the previous graph to see the details of the excitations. The reference coupling is $R_{0}=0.05$ Ns/m. The color legends pertain to the relevant coupling parameter *R*.

For better visibility, we made an enlarged version of the previous Figure 13. The zoomed-in Figure 14 shows an interesting trench at ω∼4 1/s. All of this is presumably a consequence of the nonlinear coupling. We remark that we do not need to normalize the spectra. Since the initial conditions are the same for all calculations, the total energy is constant as the form of Hamiltonian shows in Equation (40). The Wilberforce system is conservative, the energy is distributed along all the appearing frequencies.

We see that as the coupling strength increases, more and more modes appear. Since the condition for the coherence of two vibrations is the same frequency, it is quite clear that a loss of coherence occurs. This occurs even though the basic system also contains several vibration modes. What happens here now is that additional modes appear. Therefore, we can speak about decoherence and chaotic behavior in the relationship between the two systems.

We start from the fact that it is not essential what additional mode appears since if the frequency is different, it is enough to lose coherence. For physical reasons, it is otherwise obvious that modes close to the normal frequency appear. It would be strange if a large excitation appeared for a small change in coupling strength. At the same time, this is not excluded in the event of a major change, as can be seen in Figure 11.

**Figure 15:**
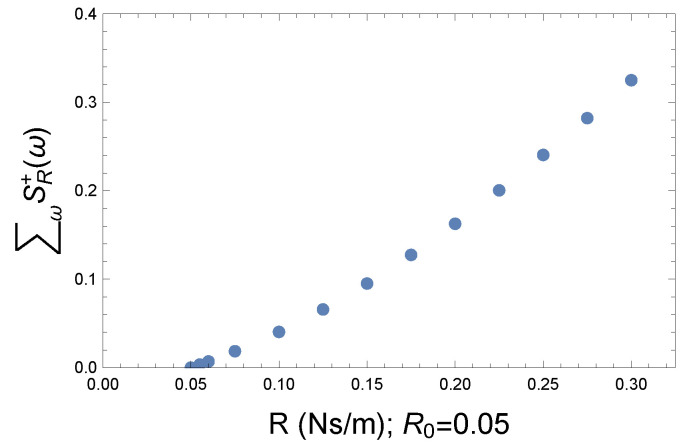
The defined loss of coherence compared to the reference system with the coupling parameter $R_{0}=0.05$ Ns/m. The decoherence relates to the measure of signal distortion or dissipation.

In Figure 13 the spectrum values, $S_{R}^{+}\left(\omega \right)$, are proportional to the occurrence of a given frequency. This figure also clearly shows that, in addition to the decrease in the occurrence of the frequencies belonging to the normal mode, the occurrence of the other frequencies increases. We can calculate a sum, $\Sigma_{\omega }S_{R}^{+}\left(\omega \right)$, which includes the contribution from all frequencies. Mathematically, this is equivalent to performing a numerical integration over the range of frequencies. Plotting these sums for different coupling values in Figure 15, we can see the trend is monotonically increasing. As a consequence of the increasing nonlinear coupling, more and more modes other than the natural frequency appear. This in itself characterizes the loss of coherence.

**Figure 16:**
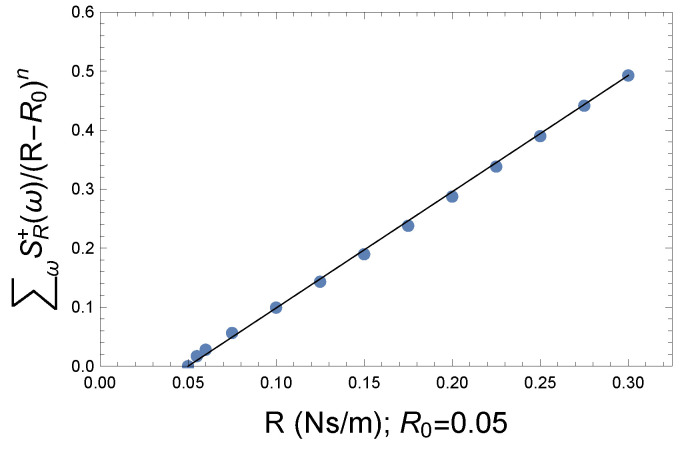
The linearized plot of the defined loss of coherence comparing to the reference system with the coupling parameter $R_{0}=0.05$ Ns/m. The plot in Figure 15 turns into a linear dependence applying the $1/{\left(R-R_{0}\right)}^{n}$ factor. The linearization parameter is n∼0.3.

It is worth making this otherwise smooth curve linear, not because it shows the trend better but because the operation may contain a characteristic value. To find the function in question, we assume that it is not necessarily complicated and must be related to the strength of the couplings. Multiplication by a factor $1/{\left(R-R_{0}\right)}^{n}$ seems to be a suitable choice for linearization. Here, $R_{0}$ and *R* are the coupling strength, while the parameter *n* is to be determined during the fitting. The straight line calculated with a good approximation is shown in Figure 16.

The characteristic parameter obtained is n∼0.3. The slope of the linear function is approximately 1.97. Both the slope of the line and the parameter *n* are related to the rate of appearance of the further excitation frequencies. Since these frequencies are the primary cause of coherence loss, it is technically better if the slope is smaller. Fewer additional frequencies means less energy dissipation. This is true whether we take it in the classical or quantum sense. This statement also holds true in a thermodynamic sense. When the dissipated signal reaches the same point as the smaller coupling, it will not be coherent again, so the slope of the line is also a measure of irreversibility.

## 10. Summary

This article examines the motion of a chain that has a magnetic moment. In addition to the Larmor precession movement, the built model also includes the interaction between magnetic moments, which leads to longitudinal oscillation. The mathematical construction leads to the Wilberforce pendulum family and the difference appears in the nonlinear coupling. However, this nonlinear behavior implies substantial changes in the solutions. Even a small change in the strength of the coupling leads not only to a shift in the normal modes but to the appearance of new excitations. This means that if the coupling is greater at a given point in the traveling wave, many modes not found in the original wave appear. This leads to distortion or loss in the initial starting coherent state.

That is why it is advisable to formulate a numerical framework to quantify the degree of coherence loss and chaotic behavior. We present a procedure that can be used to express these numerical values. We consider the spectrum of the initial wave (state) as a basis and see what modes appear in comparison to the spectrum in the case of a slightly larger coupling. From the comparison of the two spectra, we consider only those modes that have newly appeared. We show that as the coupling strength increases, a strict monotonic increase in the appearance of new modes is found. At the same time, the trend can be represented as a smooth function. A suitable factor can be used to derive a linear relationship. All this means that the degree of coherence loss and chaoticity can be expressed using a numerical value. The meaning behind this idea is that we have a mathematical picture of the amount of losses during signal transmission and reconstruction.

## 11. Conclusions

This study demonstrates that the Wilberforce-like oscillations of magnetic moments, particularly in spin chains, can lead to the emergence of additional vibrational modes due to the nonlinear coupling between translational vibrations and magnetic moment precession. These additional modes contribute to a loss in coherence and signal dissipation, which are influenced by the strength of the coupling. The findings emphasize the importance of these effects in understanding signal propagation and preservation. Moreover, the study provides a mathematical framework to quantify decoherence and dissipation, offering insights into the irreversibility of these processes.

## Data Availability

Data are contained within the article.
